# USABILITY OF A SMARTPHONE APP DESIGNED TO COLLECT HEALTH INFORMATION IN OLDER ADULTS

**DOI:** 10.1093/geroni/igad104.3685

**Published:** 2023-12-21

**Authors:** Joanne Murabito, Jamie Faro, Yuankai Zhang, Angelo Demalia, Alexander Hamel, Nakesha Agyapong, David McManus, Belinda Borrelli

**Affiliations:** Boston University Chobanian & Avedisian School of Medicine, Boston, Massachusetts, United States; University of Massachusetts Chan Medical School, Boston, Massachusetts, United States; Boston University School of Public Health, Boston, Massachusetts, United States; Boston University, Boston, Massachusetts, United States; University of Massachusetts Chan Medical School, Boston, Massachusetts, United States; University of Massachusetts Chan Medical School, Boston, Massachusetts, United States; University of Massachusetts Chan Medical School, Worcester, Boston, Massachusetts, United States; Boston University, Henry M. Goldman School of Dental Medicine, Boston, Massachusetts, United States

## Abstract

Few studies evaluate the usability of mobile-phone assessments in older adults. The aim of this study was to identify design-based barriers and facilitators to mobile app survey completion among two samples of older adults; those in the Framingham Heart Study (n=15, mean age 72 years, 40% women, 100% non-Hispanic, White) and, to aid with generalization, a more diverse sample (n=15, mean age 71 years, 47% women, 53% non-White, 20% Hispanic). We used mixed methods to ascertain information about challenging or beneficial features and functions of the mobile app. A variety of measures with different response formats were tested including self-reported surveys, pictorial format assessments (image of a body with checkboxes to indicate pain sites), and cognitive testing tasks, including Trail Making Test (TMT) and Stroop. Participants completed each of the measures using a think-aloud task, while being audio- and video-recorded. Recordings were coded for usability errors by two pairs of coders. Participants completed the Mobile App Rating Scale (MARS) to assess functionality and aesthetics of the app (range 1-5). In general, participants were satisfied with the app. In Framingham Heart Study participants, the average MARS functionality score was 3.6 (SD=0.7) and aesthetics score was 3.9 (SD=0.7) with no significant differences in the diverse sample. The majority of older adults did not have difficulty completing the self-report measures, unless there were lengthy instructions or the pictorial measure, but participants had usability issues with the Stroop and TMT. Our methods and results can guide app development and survey construction for older adults.

